# Colossal Kerr nonlinearity without absorption in a five-level atomic medium

**DOI:** 10.1038/s41598-023-51134-9

**Published:** 2024-01-18

**Authors:** Nguyen Huy Bang, Le Van Doai

**Affiliations:** Vinh University, 182 Le Duan Street, Vinh City, Vietnam

**Keywords:** Materials science, Optics and photonics

## Abstract

In this work, we present an analytical method to achieve giant Kerr nonlinearity without absorption in a five-level atomic medium. By using iterative perturbation technique on density matrix equations, we have derived the analytical expressions of nonlinear susceptibility and Kerr nonlinear coefficient in the presence of spontaneously generated coherence (SGC) and relative phase between applied laser fields. It shows that, this five-level atomic medium exhibits multiple electromagnetically induced transparency (EIT) at three different frequencies, at the same time, the Kerr nonlinear coefficient is enhanced around three transparent spectral regions; in each such EIT region appears a pair of positive–negative peaks of Kerr nonlinear coefficient. In particular, these nonlinear peaks are moved to the center of the EIT windows via SGC. This means that the Kerr nonlinear coefficient is enhanced with completely suppressed absorption at different transparency frequencies. Furthermore, the magnitude and the sign of the Kerr nonlinear coefficient are easily controlled according to the SGC strength, the coupling laser intensity, and the relative phase between applied laser fields. Such a giant nonlinear medium can be useful for photonic devices working in the resonant frequency region without absorption. As a typical application, this giant Kerr nonlinear material has been applied to an interferometer for the formation of optical bistability, and showed the appearance of OB at the resonant frequency with significantly reduced threshold intensity and OB width.

## Introduction

As we known that, Kerr nonlinear materials play a very important role in photonic devices such as optical bistability, optical switches, optical memory, logic gates, four-wave mixing, optical solitions, etc.,^1^. However, most optical materials exhibit extremely weak nonlinearity, necessitating high-intensity light sources to induce nonlinear optical phenomena. Of course, in the atomic resonance region the nonlinearity can significantly enhanced, but the light signal is also strongly absorbed by the atomic medium. Therefore, the search for large nonlinear materials is essential to be able to observe nonlinear optical effects with low intensity lights. In the past decades, the discovery of electromagnetically induced transparency (EIT)^2^ has yielded an excellent method to obtain the giant Kerr nonlinearity of the atomic medium in the vicinity of atomic resonance frequency with reduced absorption^3,4^. Indeed, Wang et al.^4^ have theoretically and experimentally demonstrated the giant enhancement of Kerr nonlinear coefficient in a three-level atomic medium under EIT condition. They then used this EIT material to create the OB effect with very low threshold intensity^5^. In addition, in the presence of EIT the magnitude and the sign of the Kerr nonlinearity can be changed by adjusting the intensity or the frequency of the laser beams^4,6^, so that the nonlinear optical phenomena are also easily manipulated by the external fields^7^.

Besides, current interests are focused on multi-level atomic media with multiple transparency frequencies occurring at different atomic transitions, and hence the Kerr nonlinearity can also be enhanced at various transparency frequencies^8–12^. In general, to achieve multi-window EIT it is necessary to use several coupling laser fields (along with a probe laser field)^13^. More simply, we can use a single coupling laser field to excite several closely spaced hyperfine levels. In particular, the five-level cascade-type configuration of the ^85^Rb atom is considered a typical model for multi-EIT generation of hyperfine levels. Wang et al.^14^ experimentally observed EIT spectrum of five-level cascade-type ^85^Rb atom, revealing three EIT windows. Theoretically, we have presented an analytical method for electromagnetically induced transparency in this five-level atomic system^15^ and subsequently developed the model to study the enhancement of Kerr nonlinearity^16–20^. It is demonstrated that the Kerr nonlinearity is basically modified and greatly enhanced around three frequency regions corresponding to three EIT windows. Furthermore, the magnitude and the sign of the Kerr nonlinear coefficient can be controlled by adjusting the intensity and the frequency of the applied laser fields. These analytical models have been employed to generate multi-frequency OB effect^21^ and to fit the experimental EIT spectra of multi-level atomic systems^22,23^.

Despite the giant nonlinearity exhibited by EIT materials near transparent spectral region (called as EIT window), the nonlinear coefficient is still zero at the center of the EIT window (where absorption is zero)^4,16^. To address this limitation, several studies^24–26^ have utilized spontaneously generated coherence (SGC) to shift the enhanced nonlinear peaks towards the center of the EIT window, enabling maximum nonlinearity with completely suppressed absorption. This approach allows nonlinear optical effects to occur with single photon. As we know that, SGC is the quantum interference effect arising from the spontaneous emission processes in atomic or molecular system with nonorthogonality of electric dipole moments induced by coherent light fields^27^. It also profoundly modifies the optical response of the atomic medium without destroying the EIT effect^28^. The influence of SGC on the optical properties of three-level atomic systems has been basically investigated for absorption and dispersion^28,29^, group velocity^30,31^, optical bistability^32,33^ and pulse propagation^34^. It was shown that the SGC can be used as a “knob” to control the optical properties of the atomic medium. Moreover, the responses of the atomic system with SGC are highly sensitive to the relative phase of the applied laser fields^30,32^. Regarding the nonlinear optical property, Niu et al.^24^ first derived expressions for the Kerr nonlinearity of three-level atomic systems in the presence of SGC and achieved giant Kerr nonlinearity with zero absorption via SGC. In addition, with the presence of the SGC effect, the magnitude of Kerr nonlinear coefficient also depends on the relative phase between the applied laser fields^35^. However, these studies only achieve Kerr nonlinearity enhancement at one transparent spectral region, resulting in only one pair of negative–positive values of the nonlinear coefficient emerging in the EIT window at the resonance frequency. Very recently, the influence of SGC on the linear optical properties including absorption, dispersion and group index in the five-level atomic system has also presented^36^. It showed that all three EIT windows of system become deeper and narrower as the SGC strength increases, leading to an increase in the slope and the amplitude of the dispersion and group index curves. However, there is still a lack of a Kerr nonlinear model of a multi-level atomic system in the presence of SCG and laser phase that can achieve giant Kerr nonlinearities at many resonant frequencies with suppressed absorption.

In this work, using an iterative method we derive the density matrix solutions up to third-order perturbation, and the expression for Kerr nonlinear coefficient of the five-level atomic system under SGC and the relative phase. We then investigate the influence of SGC and relative phase on Kerr nonlinearity to achieve giant Kerr nonlinearity at multiple light frequencies with zero absorption, which can be useful for photonic devices working at low intensity lights. As a typical application, we apply this material to an interferometer for the formation of optical bistability at the atomic resonance frequency.

## Theoretical model

**Figure 1:**
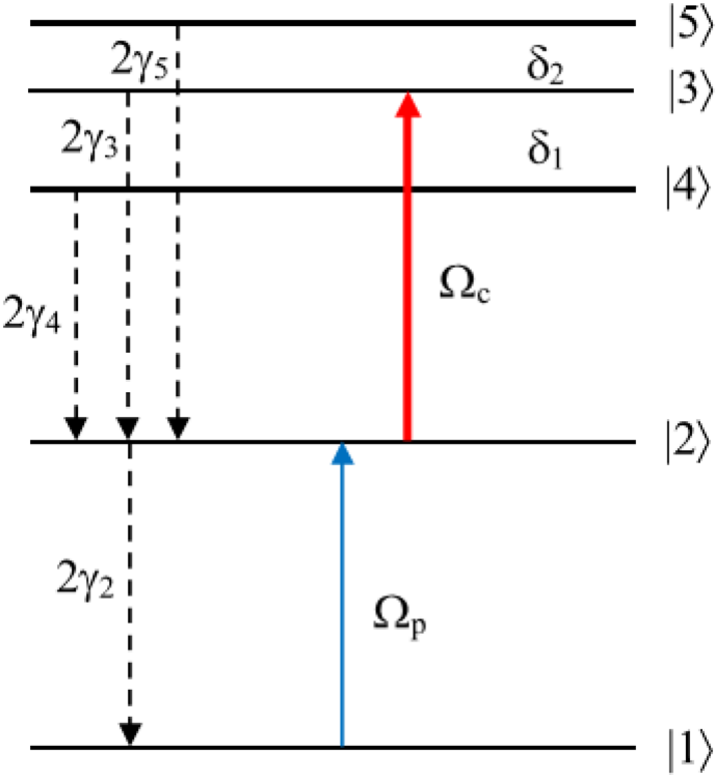
Schematic diagram of five-level cascade-type atomic system.

Figure 1 shows a five-level atomic diagram of cascade-type configuration that excited by a probe laser field and only one coupling laser field. The weak probe laser field is applied to the transition |1⟩ ↔|2⟩, while the intense coupling laser field is coupled simultaneously three transitions |2⟩ ↔|3⟩, |2⟩ ↔|4⟩ and |2⟩ ↔|5⟩. The spontaneous decay rates of the upper level |*i*⟩ to the lower level |*k*⟩ are denoted by 2*γ*_*i*_. We define $\Omega_{p} = d_{21} E_{p} /2\hbar$ and $\Omega_{c} = d_{32} E_{c} /2\hbar$ are Rabi frequency of the probe and coupling fields, respectively, with *d*_*ik*_ represents the dipole moment of the |*i*⟩ ↔|*k*⟩ transition. The coupling strengths between the level |2⟩ with three hyperfine levels |3⟩, |4⟩ and |5⟩ are given by $a_{32} = d_{32} /d_{32}$, $a_{42} = d_{42} /d_{32}$, and $a_{52} = d_{52} /d_{32}$. The frequency detunings of the probe and coupling lasers from the atomic resonant frequencies are respectively defined as $\Delta_{p} = \omega_{21} - \omega_{p}$ and $\Delta_{c} = \omega_{32} - \omega_{c}$. Let φ_p_ and φ_c_ be the phase of the probe and coupling fields, then φ = φ_p_–φ_c_ is the relative phase between the probe and coupling fields.

The evolution of the atomic states in the applied laser fields obeys the Liouville equation as follows:

1 $$ \dot{\rho } = - \frac{i}{\hbar }\left[ {H,\rho } \right] + \wedge \rho , $$

where *H* is the total Hamiltonian which can be written in the interaction picture as:

2 $$ H = \hbar \left( {\begin{array}{*{20}c} 0 & {\Omega_{p} e^{{ - i\varphi_{p} }} } & 0 & 0 & 0 \\ {\Omega_{p} e^{{i\varphi_{p} }} } & {\Delta_{p} } & {\Omega_{c} e^{{ - i\varphi_{c} }} a_{32} } & {\Omega_{c} e^{{ - i\varphi_{c} }} a_{42} } & {\Omega_{c} e^{{ - i\varphi_{c} }} a_{52} } \\ 0 & {\Omega_{c} e^{{i\varphi_{c} }} a_{32} } & {\Delta_{p} + \Delta_{c} } & 0 & 0 \\ 0 & {\Omega_{c} e^{{i\varphi_{c} }} a_{42} } & 0 & {\Delta_{p} + \Delta_{c} + \delta_{1} } & 0 \\ 0 & {\Omega_{c} e^{{i\varphi_{c} }} a_{52} } & 0 & 0 & {\Delta_{p} + \Delta_{c} - \delta_{2} } \\ \end{array} } \right), $$

In the presence of the SGC, the relaxation operator ${\Lambda }\rho$ is given by^37^:

$$ \begin{aligned} \Lambda \rho & = - \gamma_{2} (S_{2}^{ + } S_{2}^{ - } \rho + \rho S_{2}^{ + } S_{2}^{ - } - 2S_{2}^{ - } \rho S_{2}^{ + } ) - \gamma_{3} (S_{3}^{ + } S_{3}^{ - } \rho + \rho S_{3}^{ + } S_{3}^{ - } - 2S_{3}^{ - } \rho S_{3}^{ + } ) \\ & \quad - \gamma_{4} (S_{4}^{ + } S_{4}^{ - } \rho + \rho S_{4}^{ + } S_{4}^{ - } - 2S_{4}^{ - } \rho S_{4}^{ + } ) - \gamma_{5} (S_{5}^{ + } S_{5}^{ - } \rho + \rho S_{5}^{ + } S_{5}^{ - } - 2S_{5}^{ - } \rho S_{5}^{ + } ) \\ & \quad - \sqrt {\gamma_{2} \gamma_{3} } (S_{2}^{ + } S_{3}^{ - } \rho + \rho S_{2}^{ + } S_{3}^{ - } - 2S_{3}^{ - } \rho S_{2}^{ + } ) - \sqrt {\gamma_{2} \gamma_{3} } (S_{3}^{ + } S_{2}^{ - } \rho + \rho S_{3}^{ + } S_{2}^{ - } - 2S_{2}^{ - } \rho S_{3}^{ + } ) \\ & \quad - \sqrt {\gamma_{2} \gamma_{4} } (S_{2}^{ + } S_{4}^{ - } \rho + \rho S_{2}^{ + } S_{4}^{ - } - 2S_{4}^{ - } \rho S_{2}^{ + } ) - \sqrt {\gamma_{2} \gamma_{3} } (S_{4}^{ + } S_{2}^{ - } \rho + \rho S_{4}^{ + } S_{2}^{ - } - 2S_{2}^{ - } \rho S_{4}^{ + } ) \\ & \quad - \sqrt {\gamma_{2} \gamma_{5} } (S_{2}^{ + } S_{5}^{ - } \rho + \rho S_{2}^{ + } S_{5}^{ - } - 2S_{5}^{ - } \rho S_{2}^{ + } ) - \sqrt {\gamma_{2} \gamma_{5} } (S_{5}^{ + } S_{2}^{ - } \rho + \rho S_{5}^{ + } S_{2}^{ - } - 2S_{2}^{ - } \rho S_{5}^{ + } ) \\ & = \left( {\begin{array}{*{20}l} {2\rho_{22} \gamma_{2} } \hfill & { - \rho_{12} \gamma_{2} + 2p(\rho_{23} \sqrt {\gamma_{2} \gamma_{3} } + 2\rho_{24} \sqrt {\gamma_{2} \gamma_{4} } + 2\rho_{25} \sqrt {\gamma_{2} \gamma_{5} } )} \hfill \\ { - \rho_{21} \gamma_{2} + 2p(\rho_{32} \sqrt {\gamma_{2} \gamma_{3} } + 2\rho_{42} \sqrt {\gamma_{2} \gamma_{4} } + 2\rho_{52} \sqrt {\gamma_{2} \gamma_{5} } )} \hfill & { - 2\rho_{22} \gamma_{2} + 2\rho_{33} \gamma_{3} + 2\rho_{44} \gamma_{4} + 2\rho_{55} \gamma_{5} } \hfill \\ { - \rho_{31} \gamma_{3} } \hfill & { - \rho_{32} (\gamma_{2} + \gamma_{3} )} \hfill \\ { - \rho_{41} \gamma_{4} } \hfill & { - \rho_{42} (\gamma_{2} + \gamma_{4} )} \hfill \\ { - \rho_{51} \gamma_{5} } \hfill & { - \rho_{52} (\gamma_{2} + \gamma_{5} )} \hfill \\ \end{array} } \right. \\ \end{aligned} $$

3 $$ \left. {\begin{array}{*{20}l} { - \rho_{13} \gamma_{3} } \hfill & { - \rho_{14} \gamma_{4} } \hfill & { - \rho_{15} \gamma_{5} } \hfill \\ { - \rho_{23} (\gamma_{2} + \gamma_{3} )} \hfill & { - \rho_{24} (\gamma_{2} + \gamma_{4} )} \hfill & { - \rho_{25} (\gamma_{2} + \gamma_{5} )} \hfill \\ { - 2\rho_{33} \gamma_{3} } \hfill & { - \rho_{34} (\gamma_{3} + \gamma_{4} )} \hfill & { - \rho_{35} (\gamma_{3} + \gamma_{5} )} \hfill \\ { - \rho_{43} (\gamma_{3} + \gamma_{4} )} \hfill & { - 2\rho_{44} \gamma_{4} } \hfill & { - \rho_{45} (\gamma_{4} + \gamma_{5} )} \hfill \\ { - \rho_{53} (\gamma_{3} + \gamma_{5} )} \hfill & { - \rho_{54} (\gamma_{4} + \gamma_{5} )} \hfill & { - 2\rho_{55} \gamma_{5} } \hfill \\ \end{array} } \right), $$

here, $S_{2}^{ + } = \left| 2 \right\rangle \left\langle 1 \right|$, $S_{2}^{ - } = \left| 1 \right\rangle \left\langle 2 \right|$, $S_{3}^{ + } = \left| 3 \right\rangle \left\langle 2 \right|$, $S_{2}^{ - } = \left| 2 \right\rangle \left\langle 3 \right|$, $S_{4}^{ + } = \left| 4 \right\rangle \left\langle 2 \right|$, $S_{4}^{ - } = \left| 2 \right\rangle \left\langle 4 \right|$ and $S_{5}^{ + } = \left| 5 \right\rangle \left\langle 2 \right|$, $S_{5}^{ - } = \left| 2 \right\rangle \left\langle 5 \right|$ are symmetric and antisymmetric superpositions of the dipole moments, respectively; and *ρ* is a 5 × 5 matrix in which the matrix element in the *i*th row and the *j*th column is equal to 1, and the rest is zero^36^.

From Eqs. (1)–(3), the density matrix equations involving the SGC and the relative phase can be derived under the dipole and rotating-wave approximations as:

4a $$ \dot{\rho }_{55} = - 2\gamma_{5} \rho_{55} + i\Omega_{c} a_{52} (\rho_{25} - \rho_{52} ), $$

4b $$ \dot{\rho }_{44} = - 2\gamma_{4} \rho_{44} + i\Omega_{c} a_{42} (\rho_{24} - \rho_{42} ), $$

4c $$ \dot{\rho }_{33} = - 2\gamma_{3} \rho_{33} + i\Omega_{c} a_{32} (\rho_{23} - \rho_{32} ), $$

4d $$ \begin{aligned} \dot{\rho }_{22} & = - 2\rho_{22} \gamma_{2} + 2\rho_{33} \gamma_{3} + 2\rho_{44} \gamma_{4} + 2\rho_{55} \gamma_{5} - i\Omega_{p} (\rho_{21} - \rho_{12} ) \\ & \quad - i\Omega_{c} a_{32} (\rho_{23} - \rho_{32} ) - i\Omega_{c} a_{42} (\rho_{24} - \rho_{42} ) - i\Omega_{c} a_{52} (\rho_{25} - \rho_{52} ), \\ \end{aligned} $$

4e $$ \dot{\rho }_{11} = 2\gamma_{2} \rho_{22} + i\Omega_{p} (\rho_{21} - \rho_{12} ), $$

4f $$ \begin{aligned} \dot{\rho }_{21} & = - \gamma_{21} \rho_{21} + 2pe^{i\varphi } \left( {\sqrt {\gamma_{2} \gamma_{3} } \rho_{32} + \sqrt {\gamma_{2} \gamma_{4} } \rho_{42} + \sqrt {\gamma_{2} \gamma_{5} } \rho_{52} } \right) \\ & \quad + i\Omega_{c} (a_{32} \rho_{31} + a_{42} \rho_{41} + a_{52} \rho_{51} ) + i\Omega_{p} (\rho_{11} - \rho_{22} ), \\ \end{aligned} $$

4g $$ \dot{\rho }_{31} = - \gamma_{31} \rho_{31} - i\Omega_{p} \rho_{32} + i\Omega_{c} a_{32} \rho_{21} , $$

4h $$ \dot{\rho }_{41} = - \gamma_{41} \rho_{41} - i\Omega_{p} \rho_{42} + i\Omega_{c} a_{42}^{{}} \rho_{21} , $$

4i $$ \dot{\rho }_{51} = - \gamma_{51} \rho_{51} - i\Omega_{p} \rho_{52} + i\Omega_{c} a_{52} \rho_{21} , $$

4j $$ \dot{\rho }_{32} = - \gamma_{32} \rho_{32} - i\Omega_{p} \rho_{31} + i\Omega_{c} a_{32} (\rho_{22} - \rho_{33} ) - i\Omega_{c} a_{42} \rho_{34} - i\Omega_{c} a_{52} \rho_{35} , $$

4k $$ \dot{\rho }_{42} = - \gamma_{42} \rho_{42} - i\Omega_{p} \rho_{41} + i\Omega_{c} a_{42} (\rho_{22} - \rho_{44} ) - i\Omega_{c} a_{32} \rho_{43} - i\Omega_{c} a_{52} \rho_{45} , $$

4l $$ \dot{\rho }_{52} = - \gamma_{52} \rho_{52} - i\Omega_{p} \rho_{51} + i\Omega_{c} a_{52} (\rho_{22} - \rho_{55} ) - i\Omega_{c} a_{32} \rho_{53} - i\Omega_{c} a_{42} \rho_{54} , $$

4m $$ \rho_{11} + \rho_{22} + \rho_{33} + \rho_{44} + \rho_{55} = 1, $$

4n $$ \rho_{ki}^{{}} = \rho_{ik}^{ * } , $$

where $\gamma_{21} = \gamma_{2} + i\Delta_{p}$, $\gamma_{31} = \gamma_{3} + i(\Delta_{p} + \Delta_{c} )$, $\gamma_{41} = \gamma_{4} + i(\Delta_{p} + \Delta_{c} + \delta_{1} )$, $\gamma_{51} = \gamma_{5} + i(\Delta_{p} + \Delta_{c} - \delta_{2} )$, $\gamma_{32} = \gamma_{2} + \gamma_{3} + i\Delta_{c}$, $\gamma_{42} = \gamma_{2} + \gamma_{4} + i(\Delta_{c} + \delta_{1} )$ and $\gamma_{52} = \gamma_{2} + \gamma_{5} + i(\Delta_{c} - \delta_{2} )$, with δ_1_ and δ_2_ are the frequency gaps between the hyperfine levels |4⟩-|3⟩ and |5⟩-|3⟩, respectively. The terms $2p\sqrt {\gamma_{2} \gamma_{3} } \rho_{32}$, $2p\sqrt {\gamma_{2} \gamma_{4} } \rho_{42}$ and $2p\sqrt {\gamma_{2} \gamma_{5} } \rho_{52}$ represent the quantum interference resulting from the cross-coupling between the spontaneous emission paths |1⟩ ↔|2⟩ and (|2⟩ ↔|3⟩, |2⟩ ↔|4⟩ and |2⟩ ↔|5⟩) that called as spontaneously generated coherence (SGC); $p = \vec{d}_{21} .\vec{d}_{32} /\left| {\vec{d}_{21} } \right|\left| {\vec{d}_{32} } \right| = \cos \theta$ with *θ* is the angle between the two dipole moments. The parameter *p* represents the strength of SGC. If the two dipole moments are parallel, *p* = 1, and SGC is maximum. Whereas, if the two dipole moments are orthogonal, *p* = 0, and SGC is absent. In the presence of SGC, the Rabi frequencies are connected to the parameter *p* by the relations^31^ $\Omega_{p} = \Omega_{p0} \sqrt {1 - p^{2} } = \Omega_{p0} \sin \theta$ and $\Omega_{c} = \Omega_{c0} \sqrt {1 - p^{2} } = \Omega_{c0} \sin \theta$, where $\Omega_{p0}$ and $\Omega_{c0}$ are the value of Rabi frequencies without SGC.

Now, we solve the density-matrix equations in steady-state by using an iterative method that the density matrix elements can be written as^4^:

5 $$ \rho_{ik} = \rho_{ik}^{(0)} + \rho_{ik}^{(1)} + \rho_{ik}^{(2)} + ... + \rho_{ik}^{(r)} , $$

here, $\rho_{ik}^{(0)}$ is the initial value of density matrix when there is no external field, and $\rho_{ik}^{(r)}$ is the *r*th term that is assumed to be proportional to the *r*th power of the interaction Hamiltonian. For simplicity, we choose the coupling field is resonant to the transition |2⟩ ↔|3⟩, i.e., Δ_c_ = 0. Under the condition that the coupling field is much stronger than the probe field, the zeroth-order solution is $\rho_{11}^{(0)} \approx 1$, with other elements being zero $\rho_{22}^{(0)} \approx \rho_{33}^{(0)} \approx \rho_{44}^{(0)} \approx \rho_{55}^{(0)} \approx 0$.

Under the weak-probe approximation we obtain the first-order solution of the density matrix elements as follows:

6 $$ \rho_{21}^{(1)} = \frac{{i\Omega_{p} }}{{\gamma_{21} + \Omega_{c}^{2} \left( {\frac{{a_{32}^{2} }}{{\gamma_{31} }} + \frac{{a_{42}^{2} }}{{\gamma_{41} }} + \frac{{a_{52}^{2} }}{{\gamma_{51} }}} \right)}}. $$

7 $$ \rho_{31}^{(1)} = \frac{{ - \Omega_{p} \Omega_{c} a_{32} }}{{\gamma_{31} \left[ {\gamma_{21} + \Omega_{c}^{2} \left( {\frac{{a_{32}^{2} }}{{\gamma_{31} }} + \frac{{a_{42}^{2} }}{{\gamma_{41} }} + \frac{{a_{52}^{2} }}{{\gamma_{51} }}} \right)} \right]}}. $$

8 $$ \rho_{41}^{(1)} = \frac{{ - \Omega_{p} \Omega_{c} a_{42} }}{{\gamma_{41} \left[ {\gamma_{21} + \Omega_{c}^{2} \left( {\frac{{a_{32}^{2} }}{{\gamma_{31} }} + \frac{{a_{42}^{2} }}{{\gamma_{41} }} + \frac{{a_{52}^{2} }}{{\gamma_{51} }}} \right)} \right]}}. $$

9 $$ \rho_{51}^{(1)} = \frac{{ - \Omega_{p} \Omega_{c} a_{52} }}{{\gamma_{51} \left[ {\gamma_{21} + \Omega_{c}^{2} \left( {\frac{{a_{32}^{2} }}{{\gamma_{31} }} + \frac{{a_{42}^{2} }}{{\gamma_{41} }} + \frac{{a_{52}^{2} }}{{\gamma_{51} }}} \right)} \right]}}. $$

Using the iterative perturbation technique as in Ref.^24^ in which each successive approximation is calculated using the density matrix elements of one order less than the one being calculated, the matrix elements in the second order can be obtained as:

10 $$ \rho_{22}^{(2)} = \frac{{i\Omega_{p} (\rho_{12}^{(1)} - \rho_{21}^{(1)} )}}{{2\gamma_{2} }}. $$

11 $$ \rho_{32}^{(2)} = \frac{{\Omega_{p} (\rho_{31}^{(1)} - \rho_{13}^{(1)} )}}{{2i\gamma_{32} }} + \frac{{2i\Omega_{c} a_{32} \gamma_{3} \rho_{22}^{(2)} - i\Omega_{p} \gamma_{3} (\rho_{31}^{(1)} + \rho_{13}^{(1)} )}}{{2\left[ {\gamma_{3} \gamma_{32} + \Omega_{c}^{2} a_{32}^{2} } \right]}}. $$

12 $$ \rho_{42}^{(2)} = \frac{{\Omega_{p} (\rho_{41}^{(1)} - \rho_{14}^{(1)} )}}{{2i\gamma_{42} }} + \frac{{2i\Omega_{c} a_{42} \gamma_{4} \rho_{22}^{(2)} - i\Omega_{p} \gamma_{4} (\rho_{41}^{(1)} + \rho_{14}^{(1)} )}}{{2\left[ {\gamma_{4} \gamma_{42} + \Omega_{c}^{2} a_{42}^{2} } \right]}}. $$

13 $$ \rho_{52}^{(2)} = \frac{{\Omega_{p} (\rho_{51}^{(1)} - \rho_{15}^{(1)} )}}{{2i\gamma_{52} }} + \frac{{2i\Omega_{c} a_{52} \gamma_{5} \rho_{22}^{(2)} - i\Omega_{p} \gamma_{5} (\rho_{51}^{(1)} + \rho_{15}^{(1)} )}}{{2\left[ {\gamma_{5} \gamma_{52} + \Omega_{c}^{2} a_{52}^{2} } \right]}}. $$

14 $$ \rho_{33}^{(2)} = \frac{{i\Omega_{c} a_{32} (\rho_{23}^{(2)} - \rho_{32}^{(2)} )}}{{2\gamma_{3} }}. $$

15 $$ \rho_{44}^{(2)} = \frac{{i\Omega_{c} a_{42} (\rho_{24}^{(2)} - \rho_{42}^{(2)} )}}{{2\gamma_{4} }}. $$

16 $$ \rho_{55}^{(2)} = \frac{{i\Omega_{c} a_{52} (\rho_{25}^{(2)} - \rho_{52}^{(2)} )}}{{2\gamma_{5} }}. $$

17 $$ \rho_{31}^{(2)} = \frac{{2i\Omega_{c} a_{32} pe^{i\varphi } \sqrt {\gamma_{2} \gamma_{3} } \rho_{32}^{(2)} }}{{\gamma_{21} \gamma_{31} + \Omega_{c}^{2} a_{32}^{2} }}. $$

18 $$ \rho_{41}^{(2)} = \frac{{2i\Omega_{c} a_{42} pe^{i\varphi } \sqrt {\gamma_{2} \gamma_{4} } \rho_{42}^{(2)} }}{{\gamma_{21} \gamma_{41} + \Omega_{c}^{2} a_{42}^{2} }}. $$

19 $$ \rho_{51}^{(2)} = \frac{{2i\Omega_{c} a_{52} pe^{i\varphi } \sqrt {\gamma_{2} \gamma_{5} } \rho_{52}^{(2)} }}{{\gamma_{21} \gamma_{51} + \Omega_{c}^{2} a_{52}^{2} }}. $$

With the above procedure, the third-order matrix elements are derived as:

20 $$ \rho_{32}^{(3)} = \Omega_{p} \left[ {\frac{{(\rho_{31}^{(2)} - \rho_{13}^{(2)} )}}{{2i\gamma_{32} }}} \right.\left. { - \frac{{\gamma_{3} {[}\gamma_{2} (\rho_{31}^{(2)} + \rho_{13}^{(2)} ) + \gamma_{31} \rho_{31}^{(2)} + \gamma_{13} \rho_{13}^{(2)} {]}}}{{2i\gamma_{2} \left( {\gamma_{3} \gamma_{32} + \Omega_{c}^{2} a_{32}^{2} } \right)}}} \right]. $$

21 $$ \rho_{42}^{(3)} = \Omega_{p} \left[ {\frac{{(\rho_{41}^{(2)} - \rho_{14}^{(2)} )}}{{2i\gamma_{42} }}} \right.\left. { - \frac{{\gamma_{4} {[}\gamma_{2} (\rho_{41}^{(2)} + \rho_{14}^{(2)} ) + \gamma_{41} \rho_{41}^{(2)} + \gamma_{14} \rho_{14}^{(2)} {]}}}{{2i\gamma_{2} \left[ {\gamma_{4} \gamma_{42} + \Omega_{c}^{2} a_{42}^{2} } \right]}}} \right]. $$

22 $$ \rho_{52}^{(3)} = \Omega_{p} \left[ {\frac{{(\rho_{51}^{(2)} - \rho_{15}^{(2)} )}}{{2i\gamma_{52} }}} \right.\left. { - \frac{{\gamma_{5} {[}\gamma_{2} (\rho_{51}^{(2)} + \rho_{15}^{(2)} ) + \gamma_{51} \rho_{51}^{(2)} + \gamma_{15} \rho_{15}^{(2)} {]}}}{{2i\gamma_{2} \left[ {\gamma_{5} \gamma_{52} + \Omega_{c}^{2} a_{52}^{2} } \right]}}} \right]. $$

23 $$ \begin{aligned} \rho_{21}^{(3)} & = \frac{{\Omega_{p} \Omega_{c} \left( {\frac{{a_{32} }}{{\gamma_{31} }}\rho_{32}^{(2)} + \frac{{a_{42} }}{{\gamma_{41} }}\rho_{42}^{(2)} + \frac{{a_{52} }}{{\gamma_{51} }}\rho_{52}^{(2)} } \right)}}{{\gamma_{21} + A}} + \frac{{2pe^{i\varphi } \left( {\sqrt {\gamma_{2} \gamma_{3} } \rho_{32}^{(3)} + \sqrt {\gamma_{2} \gamma_{4} } \rho_{42}^{(3)} + \sqrt {\gamma_{2} \gamma_{5} } \rho_{52}^{(3)} } \right)}}{{\gamma_{21} + A}} \\ & \quad - \frac{{i\Omega_{p} \left( {2\rho_{22}^{(2)} + \rho_{33}^{(2)} + \rho_{44}^{(2)} + \rho_{55}^{(2)} } \right)}}{{\gamma_{21} + A}}. \\ \end{aligned} $$

where $$A = \Omega_{c}^{2} \left( {\frac{{a_{32}^{2} }}{{\gamma_{31} }} + \frac{{a_{42}^{2} }}{{\gamma_{41} }} + \frac{{a_{52}^{2} }}{{\gamma_{51} }}} \right)$$ and $$\gamma_{ki}^{{}} = \gamma_{ik}^{ * }$$.

Having the expressions of $\rho_{21}^{(1)}$ and $\rho_{21}^{(3)}$, the density matrix element $\rho_{21}^{{}}$ is determined up to third-order as $\rho_{21}^{{}} \approx \rho_{21}^{(1)} + \rho_{21}^{(3)}$. The total susceptibility of the atomic medium for the probe light field is related to the matrix element $\rho_{21}^{{}}$ by the following relation^4^:

24 $$ \chi = - \frac{{2Nd_{21}^{{}} }}{{\varepsilon_{0} E_{p} }}\rho_{21}^{{}} , $$

where *N* is the density of atoms and ε_0_ is the permittivity in a vacuum. On the other hand, the susceptibility can be expressed in the form^4^:

25 $$ \chi = \chi_{{}}^{(1)} + 3E_{p}^{2} \chi_{{}}^{(3)} . $$

By comparing Eqs. (24) and (25) the first- and third-order susceptibilities, and Kerr nonlinear coefficient can be determined by:

26 $$ \chi^{(1)} = - \frac{{2Nd_{21}^{2} }}{{\varepsilon_{0} \hbar \Omega_{p} }}\rho_{21}^{(1)} , $$

27 $$ \chi^{(3)} = - \frac{{2Nd_{21}^{4} }}{{3\varepsilon_{0} \hbar^{3} \Omega_{p}^{3} }}\rho_{21}^{(3)} , $$

28 $$ n_{2} = \frac{{3{\text{Re}} (\chi^{(3)} )}}{{4\varepsilon_{0} n_{0}^{2} c}}. $$

We note that the expression (27) represents the self-Kerr nonlinear coefficient of the five-level atomic system in the presence of SGC and the relative phase. It is a universal formula that can be reduced to the three-level ladder system^24^ by neglecting simultaneously coupling between the level |2⟩ with the levels |4⟩ and |5⟩ (when both levels |4⟩ and |5⟩ are far from the level |3⟩). This can be done by setting the coupling parameters *a*_52_ = 0, *a*_42_ = 0 in Eq. (23).

## Results and discussion

The theoretical model can be applied to ^85^Rb atoms in which the levels |1⟩, |2⟩, |3⟩, |4⟩ and |5⟩ are chosen as 5S_1/2_(*F* = 3), 5P_3/2_(*F′* = 3), 5D_5/2_(*F′′* = 2), 5D_5/2_(*F′′* = 4) and 5D_5/2_(*F′′* = 3), respectively. The atomic parameters are^14,38^: *N* = 10^8^ atoms/cm^3^, δ_1_ = 9 MHz, δ_2_ = 7.6 MHz, γ_2_ = 3 MHz, γ_3_ = γ_4_ = γ_5_ = 0.5 MHz, *d*_21_ = 1.5 × 10^–29^ C.m, and *a*_32_:*a*_42_:*a*_52_ = 1:1.46:0.6.

**Figure 2:**
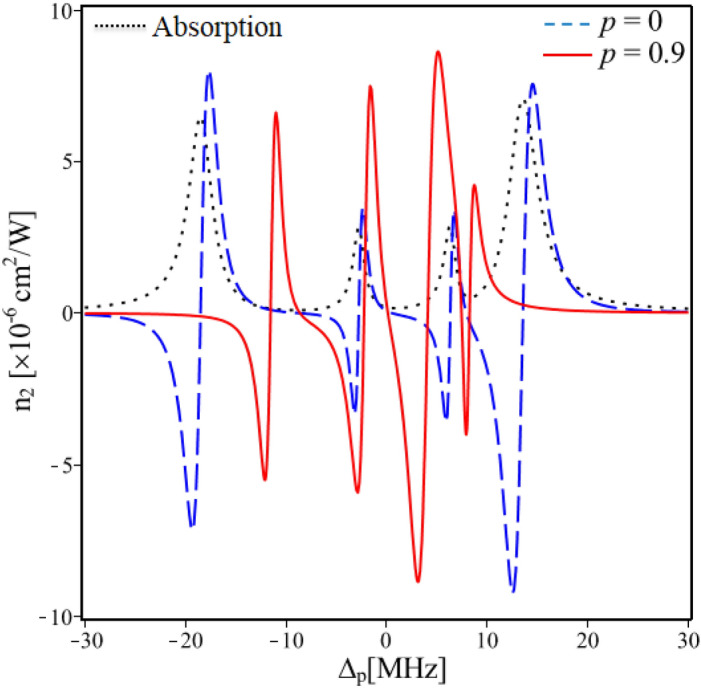
Variations of the Kerr nonlinear coefficient versus the probe detuning in the absence of SGC (dashed line) and presence of SGC with *p* = 0.9 (solid line). The dotted line is the absorption coefficient with three EIT windows at the positions Δ_p_ = 0, Δ_p_ = -9 MHz and Δ_p_ = 7.6 MHz. Other parameters are taken as Ω_c0_ = 8 MHz, Δ_c_ = 0 and φ = 0.

In order to verify the influence of the SGC on Kerr nonlinearity in this five-level atomic system, we plotted the Kerr nonlinear coefficient versus the probe detuning in the absence of SGC or* p* = 0 (dashed line) and presence of the SGC with *p* = 0.9 (solid line) as described in Fig. 2. Here, the dotted line is the absorption coefficient with three EIT windows at the positions Δ_p_ = 0, Δ_p_ = -9 MHz and Δ_p_ = 7.6 MHz^15^. Figure 2 shows that when *p* = 0, the Kerr nonlinearity (*n*_2_) is remarkably enhanced around three transparent windows in which there is a pair of positive–negative peaks of *n*_2_ emerges in each transparent window^16^. However, at the enhanced nonlinear peaks, there is still strong probe absorption (see the dashed line). This situation was improved when the presence of the SGC with *p* = 0.9, the Kerr nonlinear peaks are moved to the center of three EIT windows. This means that the Kerr nonlinearity is enhanced with completely suppressed absorption at multiple different frequencies (see the solid line). This can be explained as follows: the SGC effect does not destroy EIT, however, the linewidth of the absorption line becomes narrower and the absorption peaks on both sides of each EIT window also become higher than those when SGC absents^36^. These lead to the slope of the nonlinear dispersion curve is steeper and hence the nonlinear peaks are shifted to the center of the EIT windows. Such Kerr nonlinearity can be applied to photonic devices working at very low light intensities and ultrahigh sensitivities, such as optical bistability, all optical switching, slow light, diffraction grating and so on.

**Figure 3:**
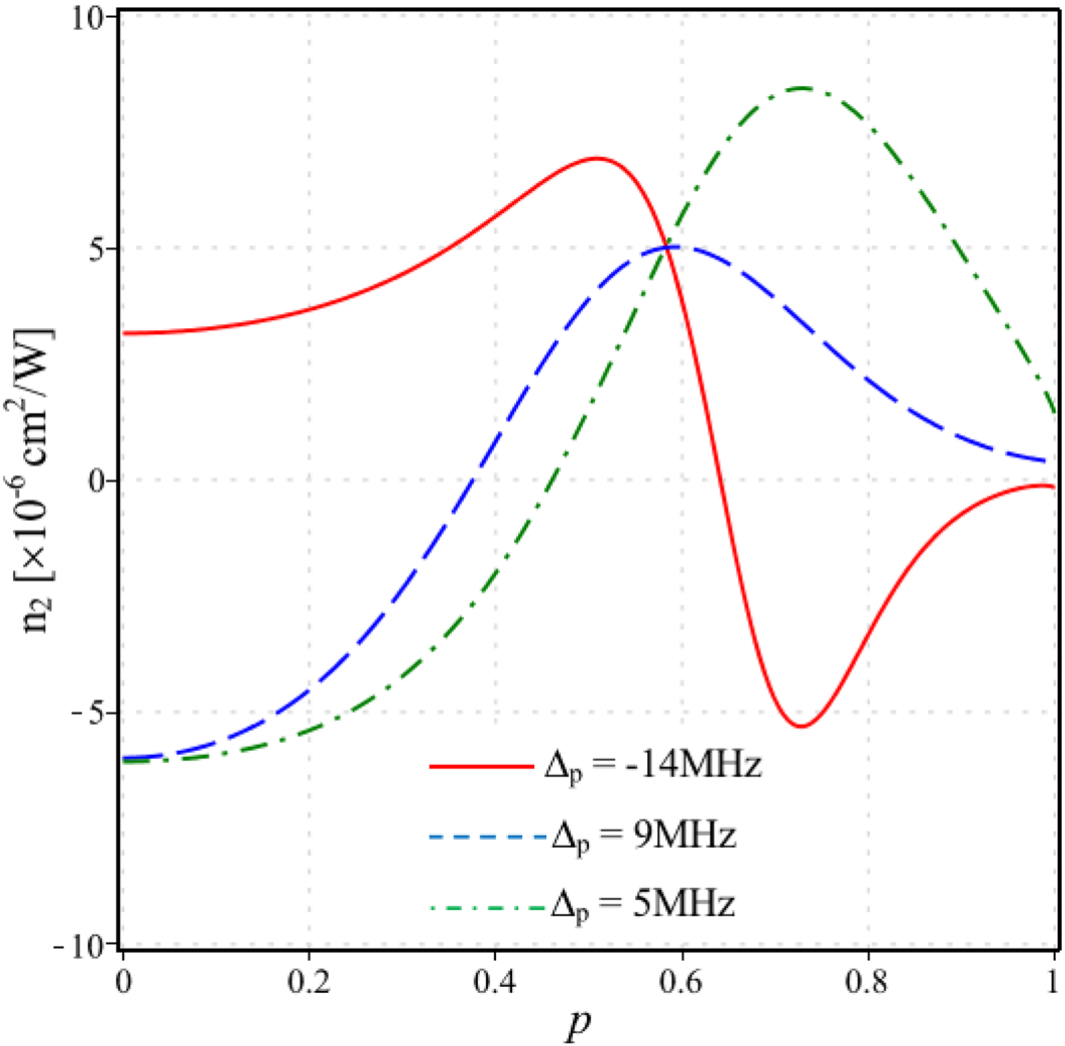
Variations of the Kerr nonlinear coefficient versus the strength of SGC *p* at different probe detunings Δ_p_ = -14 MHz (solid line), Δ_p_ = 9 MHz (dashed line) and Δ_p_ = 5 MHz (dash-dotted line). Other parameters are taken as Ω_c0_ = 8 MHz, Δ_c_ = 0 and φ = 0.

The shift of the Kerr nonlinear peaks as shown in Fig. 2, indicates that at a given probe frequency the nonlinearity of the medium is also changed when adjusting the interference parameter p. For example, in Fig. 3 we plotted the Kerr nonlinear coefficient versus the strength of SGC *p* at different probe detunings which corresponds to the enhanced nonlinear peaks at the EIT windows, Δ_p_ = − 14 MHz (solid line), Δ_p_ = 9 MHz (dashed line) and Δ_p_ = 5 MHz (dash-dotted line). It is found that the magnitude and the sign of the Kerr nonlinearity are controlled according to the strength of SGC. The Kerr nonlinear coefficient varies from positive to negative, and vice versa, when the parameter *p* increases gradually. For instance, the solid line in Fig. 3 corresponding to the selected probe frequency Δ_p_ = − 14 MHz, we can estimate the Kerr nonlinearity coefficient at several values of the parameter p as follows: n_2 _≈ 2.8 × 10^–6^ cm^2^/W at p = 0, n_2 _≈ 6.2 × 10^–6^ cm^2^/W at p = 0.5, n_2 _≈ 0 at p = 0.63, and n_2 _≈ − 5.1 × 10^–6^ cm^2^/W at p = 0.75.

**Figure 4:**
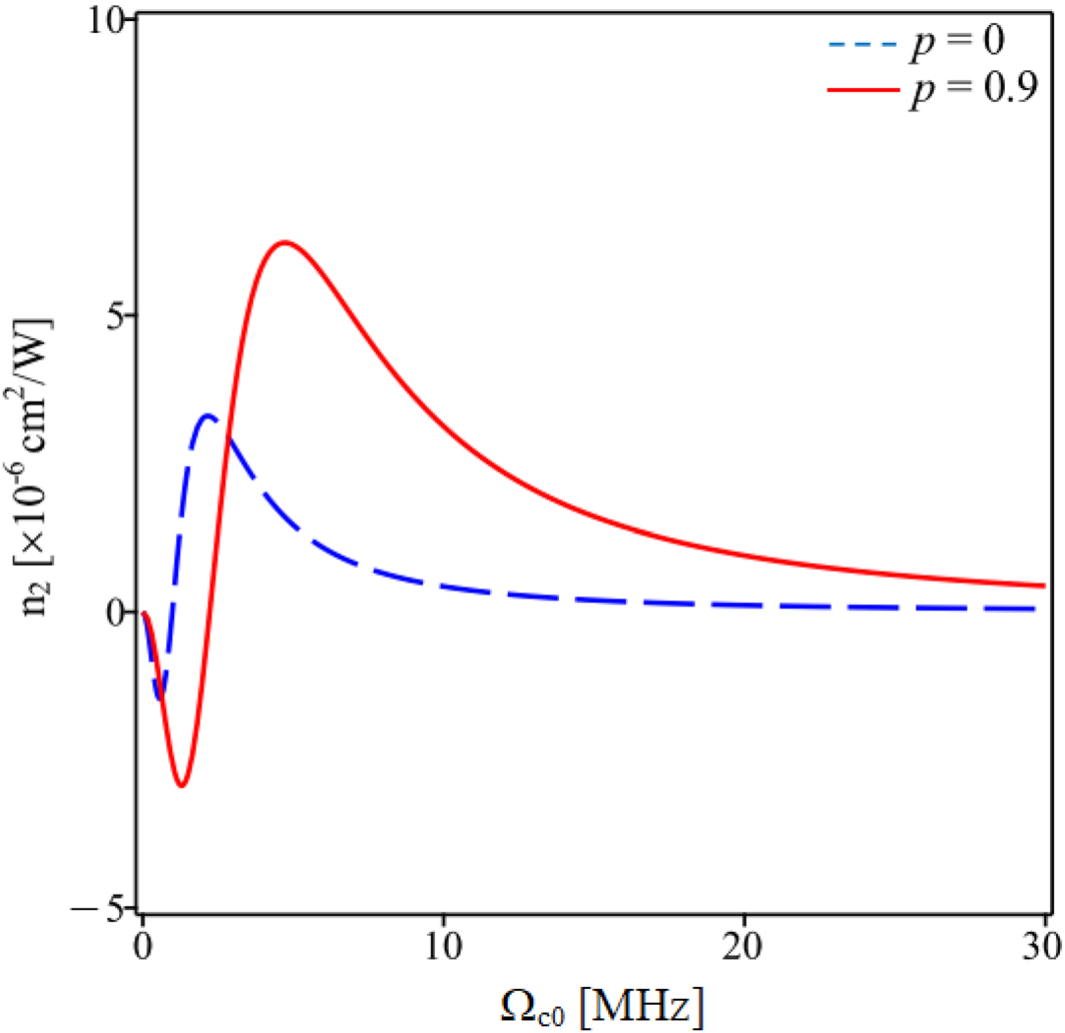
Variations of the Kerr nonlinear coefficient versus the coupling intensity Ω_c0_ for the SGC strength *p* = 0 (dashed line) and *p* = 0.9 (solid line). Other parameters are taken as Δ_p_ = Δ_c_ = 0 and φ = 0.

In Fig. 4, we investigate the variation of the Kerr nonlinear coefficient with coupling laser intensity Ω_c0_ in two cases with and without SGC. It shows that, along with the shift of the nonlinear peaks toward the center of the EIT windows by SGC, the amplitude of the nonlinear coefficient in the presence of SGC is also larger than that without SGC. This characteristic is similar to linear dispersion in that the dispersion curve (linear as well as nonlinear) becomes steeper and higher in the presence of SGC because the EIT window is narrower and deeper than the case without SGC^28,36^. In addition, when gradually increasing the coupling laser intensity, the Kerr nonlinear coefficient also quickly approaches zero in the absence of SGC, but in the case of SGC, it still retains a certain large value. We note that an increase in coupling laser intensity leads to an increase in the depth and the width of the EIT window, so that it changes the amplitude and the sign of the linear and nonlinear dispersion curves according to the Kramers–Kronig relation.

**Figure 5:**
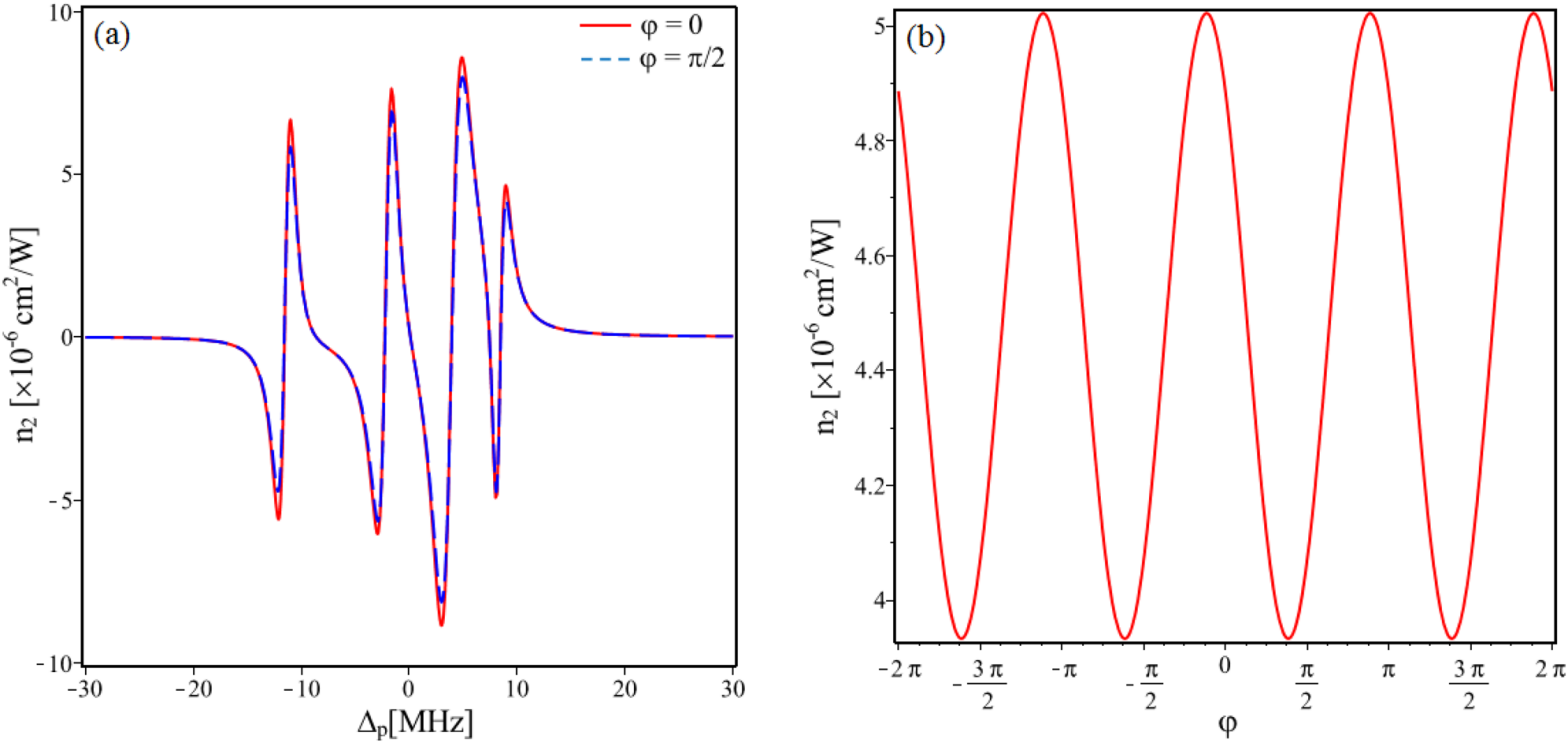
(**a**) Variations of the Kerr nonlinear coefficient versus the probe detuning when the relative phase φ = 0 (solid line) and φ = π/2 (dashed line). (**b**) Variation of the Kerr nonlinear coefficient versus the relative phase φ when the probe detuning Δ_p_ = − 2 MHz. Other parameters are taken as *p* = 0.9, Ω_c0_ = 8 MHz and Δ_c_ = 0.

In order to see the dependence of the Kerr nonlinear coefficient on the relative phase in the presence of SGC with *p* = 0.9, we plotted the Kerr nonlinear coefficient versus the probe detuning when φ = 0 (solid line) and φ = π/2 (dashed line) as displayed in Fig. 5a. At different relative phases, the amplitude of the nonlinear coefficient is also different. The variation of the Kerr nonlinear peak (at the probe frequency Δ_p_ = − 2 MHz) according to the relative phase is plotted in Fig. 5b. Thus, the magnitude of the Kerr nonlinear coefficient is moderately modulated by the relative phase with a period of 2π.

**Figure 6:**
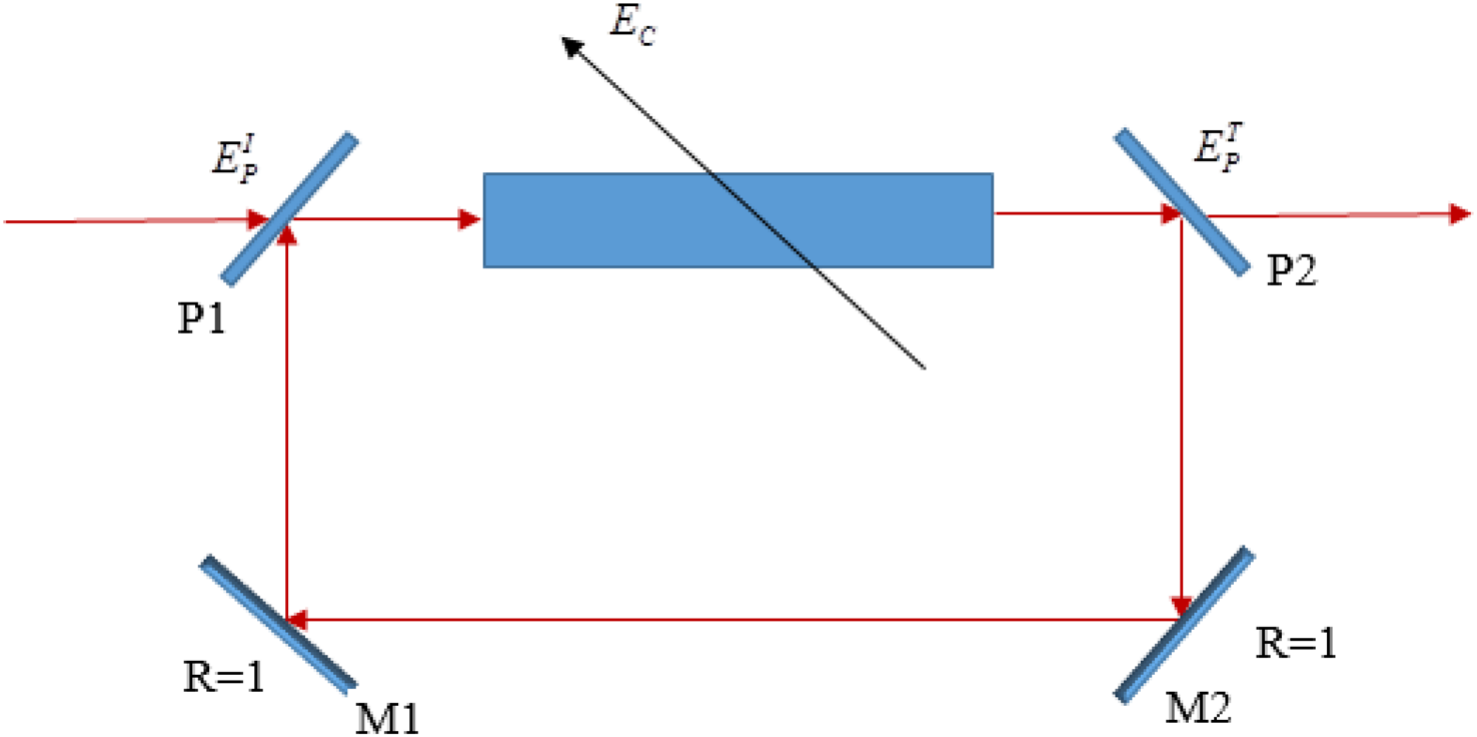
Unidirectional ring cavity containing the five-level EIT medium of length *L*; $E_{p}^{I}$ and $E_{p}^{T}$ are the incident and transmitted probe fields, respectively; $E_{c}^{{}}$ represents the coupling field which is noncirculating in the cavity.

As an example of application, we apply the colossal Kerr nonlinear material to an unidirectional ring cavity for the implementation of optical bistability, as shown in Fig. 6. In the unidirectional ring cavity, the probe field *E*_*p*_ is circulated in the cavity but nor the coupling field *E*_*c*_. The incident probe field $E_{p}^{I}$ enters through the beam splitter P_1_, interacts with the Kerr nonlinear material of the length *L*, circulates in the cavity, and partially comes out of the beam splitter P_2_ as $E_{p}^{T}$. Part of the output intensity is refracted back into the medium plays the feedback which is essential for the generation of optical bistability.

The expression for the output-input intensity of OB via Kerr nonlinear coefficient *n*_2_ is given by^1^:

29 $$ I_{out} = \left[ {\frac{1}{2} + \frac{1}{2}\cos \left( {\frac{2\pi L}{\lambda }n_{2} I_{out} + \phi } \right)} \right]I_{in} , $$

where, λ is the wavelength of the probe light, $I_{in} \sim \left( {E_{p}^{I} } \right)^{2}$ and $I_{out} \sim \left( {E_{p}^{T} } \right)^{2}$ are incident and transmitted intensities of the probe light, *n*_2_ is determined from Eq. (28), and

30 $$ \phi = \frac{2\pi L}{\lambda }n_{0} + \varphi_{p} , $$

is the round-trip phase of the probe light.

**Figure 7:**
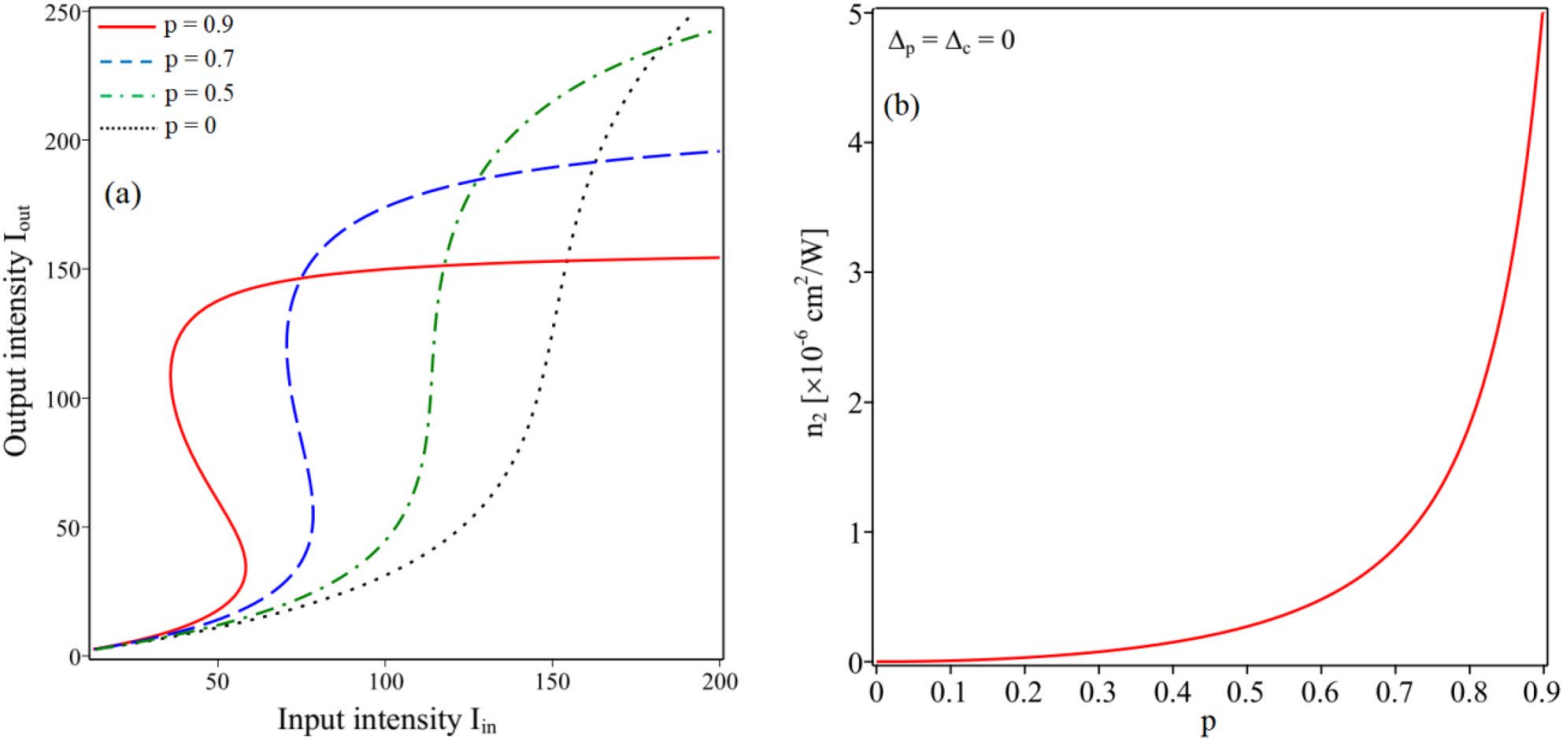
(**a**) Curves of the input–output intensity for different values of the strength of SGC, *p* = 0 (dotted line), *p* = 0.5 (dash-dotted line), *p* = 0.7 (dashed line) and *p* = 0.9 (solid line). (**b**) Variation of the Kerr nonlinear coefficient versus the strength of SGC *p* at Δ_p_ = Δ_c_ = 0. Other parameters are taken as Ω_c0_ = 8 MHz and φ = 0.

In Fig. 7a we plot the OB curves for different values of the strength of SGC *p* at two-photon resonance of the probe and coupling fields Δ_p_ = Δ_c_ = 0. From this figure, we can see that when *p* = 0, the OB effect does not appear because *n*_2_ is zero at Δ_p_ = 0, as depicted in Fig. 7b. However, when increasing the parameter *p*, the OB also gradually appears due to *n*_2_ grows with the increase of the parameter *p* (see Fig. 7b). At the same time, the threshold intensity and the width of OB are also significantly reduced (fast switching speed) when p = 0.9 due to the giant nonlinearity with zero absorption at Δ_p_ = 0 (see the solid line in Fig. 7a).

## Conclusion

In conclusion, employing the iterative perturbation technique to find solutions of density matrix elements up to the third-order perturbation, we derived the analytical expressions of nonlinear susceptibility and Kerr nonlinear coefficient in the five-level ladder-type atomic medium including spontaneously generated coherence (SGC) and relative phase between the probe and coupling laser fields. Our findings demonstrate that in the presence of SGC (with *p* = 0.9), the giant enhanced Kerr nonlinearity is achieved at atomic resonance frequencies with completely suppressed absorption. Additionally, the magnitude and the sign of the Kerr nonlinearity can be effectively controlled by adjusting the SGC strength and coupling laser intensity. Moreover, the magnitude of the Kerr nonlinear coefficient is modulated by the relative phase with a period of 2π. Along with the shift of the nonlinear peaks toward the center of the EIT windows by SGC, the amplitude of the nonlinear coefficient in the presence of SGC is also larger than that without SGC. The medium of such giant Kerr nonlinearity is applied to the interferometer for the formation of optical bistability, and showed that the OB appears in the resonant frequency region with the threshold intensity and the width being significantly reduced. Furthermore, our analytical model is also essential for the experimental observation of the Kerr nonlinear coefficient under spontaneously generated coherence. The experimental setups to study the EIT effect and Kerr nonlinearity of this model can be referred to some works^39,40^.

## Data Availability

All the data generated/analyzed during the current study available from the corresponding author on reasonable request.
